# Effect of BCG vaccination against *Mycobacterium tuberculosis* infection in adult Brazilian health-care workers: a nested clinical trial

**DOI:** 10.1016/S1473-3099(23)00818-6

**Published:** 2024-06

**Authors:** Paulo Cesar Pereira dos Santos, Nicole Louise Messina, Roberto Dias de Oliveira, Patricia Vieira da Silva, Marco Antonio Moreira Puga, Margareth Dalcolmo, Glauce dos Santos, Marcus Vinícius Guimarães de Lacerda, Bruno Araújo Jardim, Fernando Fonseca de Almeida e Val, Nigel Curtis, Jason R Andrews, Julio Croda

**Affiliations:** a Universidade Federal de Mato Grosso do Sul—UFMS, Campo Grande, Mato Grosso do Sul, Brazil; b Infectious Diseases Group, Murdoch Children's Research Institute, Parkville, VIC, Australia; c Department of Paediatrics, The University of Melbourne, Parkville, VIC, Australia; d Universidade Estadual de Mato Grosso do Sul, Dourados, Mato Grosso do Sul, Brazil; e Programa de Pós-graduação em Ciências da Saúde, Universidade Federal da Grande Dourados, Dourados, Mato Grosso do Sul, Brazil; f Centro de Referência Professor Hélio Fraga, Fundação Oswaldo Cruz, Rio de Janeiro, Brazil; g Pontifícia Universidade Católica do Rio de Janeiro, Rio de Janeiro, Brazil; h Fundação de Medicina Tropical Doutor Heitor Vieira Dourado, Manaus, Brazil; i Instituto Leônidas & Maria Deane, Oswaldo Cruz Foundation Ministry of Health, Amazonas, Brazil; j Infectious Diseases, The Royal Children's Hospital Melbourne, Parkville, VIC, Australia; k Division of Infectious Diseases and Geographic Medicine, Stanford University School of Medicine, Stanford, CA, USA; l Fiocruz Mato Grosso do Sul, Fundação Oswaldo Cruz, Campo Grande, Mato Grosso do Sul, Brazil; m Department of Epidemiology of Microbial Diseases, Yale School of Public Health, New Haven, CT, USA

## Abstract

**Background:**

The effectiveness of BCG vaccine for adult pulmonary tuberculosis remains uncertain. In this study, we aimed to evaluate the effect of vaccination with BCG-Denmark to prevent initial and sustained interferon-γ release assay conversion in Brazilian health-care workers.

**Methods:**

This substudy is a nested randomised controlled trial embedded within the BRACE trial (NCT04327206). Specifically, this substudy enrolled Brazilian health-care workers (aged ≥18 years) from three sites in Brazil (Manaus, Campo Grande, and Rio de Janeiro) irrespective of previously receiving BCG vaccination. Participants were excluded if they had contraindications to BCG vaccination, more than 1 month of treatment with specific tuberculosis treatment drugs, previous adverse reactions to BCG, recent BCG vaccination, or non-compliance with assigned interventions. Those eligible were randomly assigned (1:1) to either the BCG group (0·1 mL intradermal injection of BCG-Denmark [Danish strain 1331; AJ Vaccines, Copenhagen]) or the placebo group (intradermal injection of 0·9% saline) using a web-based randomisation process in variable-length blocks (2, 4, or 6), and were stratified based on the study site, age (<40, ≥40 to <60, ≥60 years), and comorbidity presence (diabetes, chronic respiratory disease, cardiac condition, hypertension). Sealed syringes were used to prevent inadvertent disclosure of group assignments. The QuantiFERON-TB Gold (QFT) Plus test (Qiagen; Hilden, Germany) was used for baseline and 12-month tuberculosis infection assessments. The primary efficacy outcome was QFT Plus conversion (≥0·35 IU/mL) by 12 months following vaccination in participants who had a negative baseline result (<0·35 IU/mL).

**Findings:**

Between Oct 7, 2020, and April 12, 2021, 1985 (77·3%) of 2568 participants were eligible for QFT Plus assessment at 12 months and were included in this substudy; 996 (50·2%) of 1985 were in the BCG group and 989 (49·8%) were in the placebo group. Overall, 1475 (74·3%) of 1985 participants were women and 510 (25·7%) were men, and the median age was 39 years (IQR 32–47). During the first 12 months, QFT Plus conversion occurred in 66 (3·3%) of 1985 participants, with no significant differences by study site (p=0·897). Specifically, 34 (3·4%) of 996 participants had initial QFT conversion in the BCG group compared with 32 (3·2%) of 989 in the placebo group (risk ratio 1·09 [95% CI 0·67–1·77]; p=0·791).

**Interpretation:**

BCG-Denmark vaccination did not reduce initial QFT Plus conversion risk in Brazilian health-care workers. This finding underscores the need to better understand tuberculosis prevention in populations at high risk.

**Funding:**

Bill & Melinda Gates Foundation, the Minderoo Foundation, Sarah and Lachlan Murdoch, the Royal Children's Hospital Foundation, Health Services Union NSW, the Peter Sowerby Foundation, SA Health, the Insurance Advisernet Foundation, the NAB Foundation, the Calvert-Jones Foundation, the Modara Pines Charitable Foundation, the United Health Group Foundation, Epworth Healthcare, and individual donors.

**Translation:**

For the Portuguese translation of the abstract see Supplementary Materials section.

## Introduction

Tuberculosis remains a substantial health problem that affects millions of people and is one of the leading causes of death worldwide.^1^ Once the infection is acquired, its control relies on both the adaptive and innate immune responses, and protective immunity against *Mycobacterium tuberculosis* varies considerably between individuals.^2^, ^3^, ^4^ BCG is the only vaccine approved for the prevention of tuberculosis and has been used for more than a century.^5^, ^6^ The BCG vaccine offers protection against pulmonary, meningeal, and disseminated tuberculosis in young children, but its efficacy in adults is uncertain.^5^, ^7^, ^8^ Although new vaccines show promise in preventing tuberculosis among individuals with established infection,^9^, ^10^ the paucity of established preclinical models and human immunological markers for protection has hampered the development of a more effective vaccine.^9^, ^11^

Revaccination with BCG has been assessed as a strategy to enhance the protection against tuberculosis among adolescents and adults, but the findings have yielded mixed results.^8^ A placebo-controlled trial of repeat BCG vaccination in Malawi provided evidence of no major protection against tuberculosis,^12^ whereas a cluster-randomised repeat vaccination trial in Brazil found modest efficacy in one of two study sites.^13^ A recent placebo-controlled trial of BCG revaccination among adolescents found that revaccination did not prevent QuantiFERON-TB Gold In-tube assay (QFT) conversion; however, BCG revaccination reduced the rate of QFT sustained conversion, a secondary outcome.^11^

**Research in context**

**Evidence before this study**

Tuberculosis is a leading cause of death worldwide and remains a substantial challenge to global health, impacting millions of people. Protective immunity against *Mycobacterium tuberculosis* varies considerably from person to person. The BCG vaccine is the only approved vaccine for preventing tuberculosis. Although the BCG vaccine provides protection against pulmonary, meningeal, and disseminated tuberculosis in young children, its effectiveness in adults is uncertain. Novel vaccines have the potential to prevent tuberculosis in already infected individuals, but the paucity of preclinical models and reliable human immunological markers for protection has hindered the development of these new vaccines.

We searched PubMed for studies published in English from Aug 11, 2021, to July 7, 2023, using the search terms (“tuberculosis” OR “interferon gamma release assay” OR “QuantiFERON”) AND (“BCG” OR “Bacille Calmette-Guérin”) AND trial. We selected clinical trials that evaluated the efficacy of BCG revaccination regardless of the use of a placebo. Revaccination as a strategy to improve tuberculosis protection in adolescents and adults has shown mixed results. In a trial comparing BCG revaccination with a placebo in Malawi, no protective effect against tuberculosis was observed. A cluster-randomised vaccination study in Brazil also showed no overall tuberculosis protection, although low vaccine efficacy of 19% was observed in one site. A placebo-controlled trial in South Africa focusing on BCG revaccination in adolescents found no protection against QuantiFERON-TB Gold In-tube assay (QFT) conversion. However, revaccination did lead to a 45% reduction (p=0·03) in the secondary outcome of sustained QFT conversion. In the absence of sufficient evidence for the effectiveness of BCG in preventing pulmonary tuberculosis among adults, WHO does not recommend its use in this age group.

**Added value of this study**

Previous randomised trials found conflicting evidence for BCG revaccination in prevention of tuberculosis, with no evidence of protection in Malawi and modest protection observed in one of two sites in Brazil. In our nested randomised controlled trial across multiple centres, we did not find evidence of effectiveness of BCG vaccination against QFT conversion or sustained QuantiFERON conversion, as proxies for *M tuberculosis* infection, in Brazilian health-care workers. These findings differ from the results of a randomised trial in South Africa, which found evidence of protection against the exploratory point of sustained QFT conversion.

**Implications of all the available evidence**

The understanding of whether vaccines, including BCG, can prevent *M tuberculosis* infection is essential, acknowledging that our current tests, such as interferon-γ release assays, are imperfect proxies that reflect T-cell sensitisation. In this context, this trial did not find evidence that BCG reduces risk of QFT conversion or sustained conversion in health-care workers in a country with a high incidence of tuberculosis. These results add evidence to the uncertain role of BCG revaccination in adults for the prevention of tuberculosis, and current data do not support its use for this indication.

Because of the uncertainty of conclusive evidence for the ability of BCG vaccination in preventing pulmonary tuberculosis in adults,^8^ BCG vaccination is not recommended in this age group by WHO.^14^ In this study, we therefore aimed to evaluate the effect of vaccination with BCG-Denmark on the prevention of initial and sustained QFT Plus conversion in adult Brazilian health-care workers without previous *M tuberculosis* infection.

## Methods

### Study design and participants

This substudy is a nested randomised controlled trial that is embedded within the international, multicentre, randomised, controlled, phase 3 trial of BCG vaccination to protect health-care workers against COVID-19 (ie, the BRACE trial [NCT04327206]) done in Australia, the Netherlands, Spain, the UK, and Brazil.^15^ The BRACE trial aimed to investigate an off-target effect of the BCG vaccine in reducing the incidence and severity of COVID-19 infection compared with a placebo in this population. For this substudy, we only included participants from Brazil, because of the high tuberculosis burden compared with the other countries in the BRACE trial.^1^

This substudy involved three sites in Brazil (Campo Grande, Manaus, and Rio de Janeiro). We enrolled adult health-care workers (aged ≥18 years), irrespective of whether they had previously received BCG vaccination. Individuals were eligible for the parent trial if they worked in a health-care setting or had personal contact with patients.^16^ We included participants who consented to be contacted for future ethically approved projects and had the QFT Plus assay done at enrolment and 12 months of follow-up. Participants were excluded from the parent trial if they had any contraindication to BCG vaccination; had received more than 1 month of treatment with isoniazid, rifampicin, or quinolone; had a previous adverse reaction to the BCG vaccine (ie, substantial local reaction [abscess] or suppurative lymphadenitis); or had BCG vaccine administered in the last year.

The quality assurance of the gathered data in Brazil was done by the Clinical Research Platform of the Oswaldo Cruz Foundation (Rio de Janeiro, Brazil). The Royal Children's Hospital Melbourne (number 62586) served as the primary Human Research Ethics Committee, and the protocol received approval from all participating sites.

### Randomisation and masking

An independent statistician randomly assigned participants (1:1) to either the BCG group or placebo group, using a web-based randomisation process on the Research Electronic Data Capture platform (REDCap [versions 10.1.2 and 10.7.1]).^17^ Randomisation was in randomly permuted blocks of variable length (2, 4, or 6), and stratified by study site, age (<40, ≥40 to <60, and ≥60 years), and by the presence of comorbidity (diabetes, respiratory disease, heart condition, or hypertension).

The unmasking was restricted to those responsible for preparing and administering the intervention (BCG or placebo) and safety review. Participants, investigators, statisticians, and other trial staff who engaged in follow-up and data collection remained masked to the randomisation group. The preservation of allocation concealment was a priority, and strategies such as central randomisation and sealed syringes were used to prevent inadvertent disclosure of group assignments.

### Procedures

The REDCap platform was used for data collection. Previous BCG vaccination was ascertained by participants self reporting. Following randomisation, participants assigned to the BCG group received 0·1 mL intradermal BCG vaccine (BCG-Denmark; AJ Vaccines; Copenhagen; batch numbers 118019D and 119053A), amounting to 2–8 × 10^5^ colony forming units of *Mycobacterium bovis* (Danish strain 1331) in the deltoid muscle region. Those assigned to the placebo group received a 0·1 mL intradermal saline placebo (0·9% sodium chloride) in the deltoid muscle region.

The QFT Plus (Qiagen; Hilden, Germany) test was done on day 0 (baseline) and repeated at 12 months. A follow-up QFT Plus testing was done after a further 6 months on participants who had initial conversion by 12 months to assess whether the QFT Plus conversion was sustained. We used QFT Plus conversion as a marker of *M tuberculosis* infection to assess the protective effect of BCG vaccination compared with a placebo.

The QFT Plus test measures the amount of interferon-γ (IFN-γ) produced by cells in heparinised whole blood after exposure to specific antigens from *M tuberculosis*. The test comprises four blood collection tubes (Nil, TB1, TB2, and Mitogen). TB1 and TB2 antigen tubes have a mixture of proteins (ESAT-6 and CFP-10) to stimulate cell-mediated immune responses from CD4^+^ T-helper lymphocytes. The TB2 antigen tube has additional peptides targeted to induce a response from CD8^+^ cytotoxic T lymphocytes. The detection of IFN-γ (IU/mL) by ELISA is used to identify these responses. The Nil tube adjusts for background, and its value is subtracted from the TB1 and TB2 antigens and Mitogen IFN-γ concentrations. A Nil IFN-γ value of more than 8·0 IU/mL is interpreted as indeterminate. An assay is positive if an IFN-γ response to either TB1 or TB2 antigen is above the Nil IFN-γ value (≥0·35 IU/mL). The Mitogen tube is a positive control, and a low response (<0·5 IU/mL) indicates an indeterminate result.^18^ For this study, we considered the highest IFN-γ value produced by either antigen tube to assess conversion. This approach is consistent with the manufacturer's recommendation and interpretation of the assay results, which uses the 0·35 IU/mL threshold for either antigen tube. We additionally applied other thresholds (<0·2, ≥0·7, ≥2·0, and ≥4·0 IU/mL) based on previous studies.^19^, ^20^, ^21^

At baseline and again at 12 and 18 months, 4 mL of peripheral blood was collected for QFT Plus testing. The QFT Plus assay was done according to the manufacturer's instructions. Briefly, the blood samples collected from each participant were incubated (36–38°C for 16–24 h) for cell stimulation. After the incubation, the supernatants were harvested and stored at –80°C before they were tested in batches. ELISA was done, and the plates were read by using a microplate reader (LMR-96; Loccus; São Paulo, Brazil) fitted with a 450 nm filter and a 630 nm reference filter. Each site (Campo Grande, Manaus, and Rio de Janeiro) was responsible for processing and testing their samples and following the same protocol.

### Outcomes

The primary efficacy outcome was QFT Plus conversion (≥0·35 IU/mL) by 12 months following BCG or placebo vaccination in participants who had a negative baseline result (<0·35 IU/mL). The secondary outcome was sustained conversion, defined as two consecutive positive QFT Plus results at least 6 months apart among individuals with a baseline negative QFT.

We also evaluated the following additional exploratory outcomes: alternative QFT Plus threshold values for QFT conversion (≥0·7 IU/mL, ≥2·0 IU/mL, and ≥4·0 IU/mL); and QFT Plus conversion by 12 months in individuals with an initial QFT Plus value of 0·2 IU/mL or less. These alternative thresholds and definitions were selected based on recent studies evaluating the accuracy, stability, and predictive value of QFT Plus using these thresholds.^19^, ^20^, ^21^

### Statistical analysis

Before unmasking, on the basis of 1985 participants being enrolled and a QFT Plus conversion rate of 3·3%, we estimated a power of 78% to detect a 50% reduction in conversion for a two-sided test with an α of 0·05. The primary analysis used an intention-to-treat approach, including all randomly assigned participants according to initial allocation with a negative baseline QFT Plus result who underwent the month 12 QFT assay. We used Poisson regression models with adjustments for predefined strata (ie, site, age, and presence of comorbidity) used in the randomisation, estimating risk ratios (RRs) along with 95% CIs using sandwich estimators. We extended our evaluation to include individuals with a negative baseline QFT result, irrespective of whether they underwent the QFT Plus testing. We also did a per-protocol population analysis comprising individuals who received their allocated intervention.

In secondary analyses, we replicated these models and expanded them to incorporate additional covariates, including gender, the quantity and nature of comorbidities, and past BCG vaccination status. This extension applied to both the intention-to-treat and per-protocol populations.

We did all analyses using R (version 4.3.0). This substudy is embedded in the BRACE trial, and is registered with ClinicalTrials.gov, number NCT04327206.

### Role of the funding source

The funders of the study had no role in study design, data collection, data analysis, data interpretation, or writing of the report.

## Results

**Figure 1:**
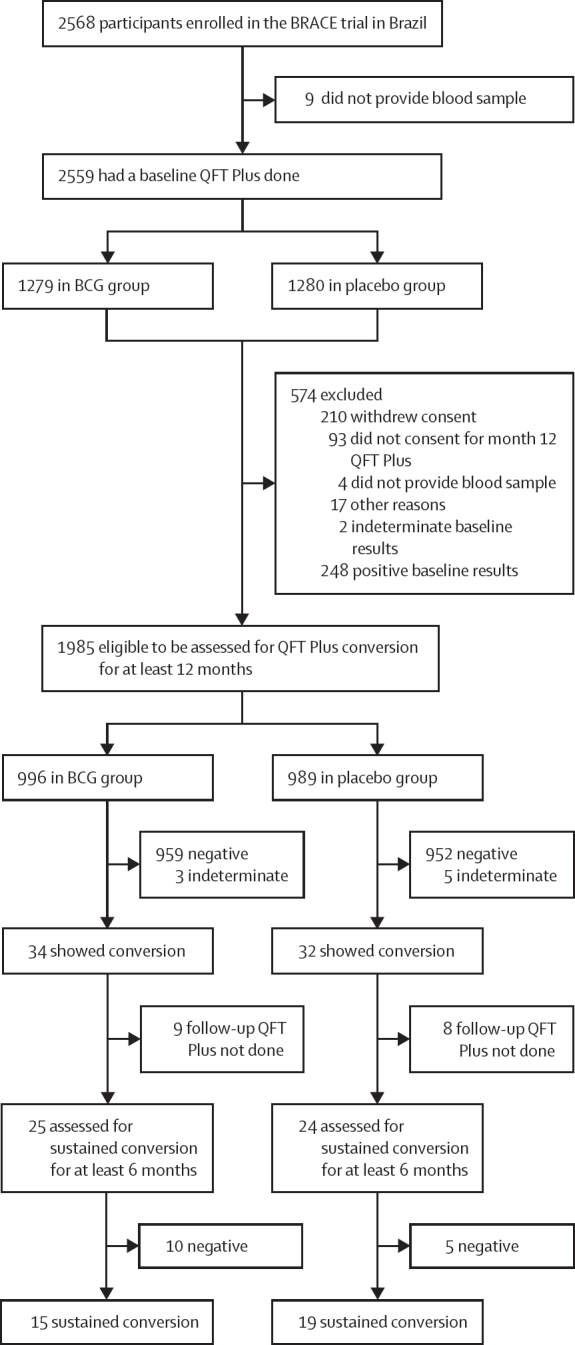
Study flowchart QFT=QuantiFERON TB Gold.

Between Oct 7, 2020, and April 12, 2021, a total of 2568 participants were enrolled in the BRACE trial in Brazil (figure 1). Nine (0·4%) of 2568 participants enrolled were excluded for not having blood samples collected at randomisation. 2559 participants had a baseline QFT Plus done, of whom 574 (22·4%) were excluded and 1985 (77·6%) were eligible to be assessed for QFT Plus conversion at 12 months. In total, 996 (50·2%) of 1985 were in the BCG group and 989 (49·8%) were in the placebo group. The baseline characteristics showed comparability between the two groups (table 1). Overall, 1475 (74·3%) of 1985 participants were women and 510 (25·7%) were men, and the median age was 39 years (IQR 32–47).

**Table 1:** Baseline demographic and clinical characteristics at baseline in the intention-to-treat population

		**BCG group (n=996)**	**Placebo group (n=989)**
Sex			
	Female	723 (72·6%)	752 (76·0%)
	Male	273 (27·4%)	237 (24·0%)
Age (years)		39·0 (31·0–47·0)	40·0 (32·0–48·0)
Coexisting condition		223 (22·4%)	228 (23·1%)
	Diabetes	35 (3·5%)	40 (4·0%)
	Chronic respiratory disease	51 (5·1%)	42 (4·2%)
	Cardiovascular disease or hypertension	137 (13·8%)	146 (14·8%)
Any alcohol use		479 (48·1%)	459 (46·4%)
Smoker		96 (9·6%)	106 (10·7%)
Previous BCG vaccination status			
	No	37 (3·7%)	40 (4·0%)
	1–5 years ago	7 (0·7%)	11 (1·1%)
	>5 years ago	952 (95·6%)	938 (94·8%)
Previous positive tuberculin skin test			
	No	948 (95·2%)	918 (92·8%)
	Not sure	23 (2·3%)	31 (3·1%)
	Yes	25 (2·5%)	40 (4·0%)
Occupation			
	Allied health	123 (12·3%)	125 (12·6%)
	Administrative or clerical	84 (8·4%)	89 (9·0%)
	Physician	36 (3·6%)	36 (3·6%)
	Nurse or midwife	97 (9·7%)	103 (10·4%)
	Other role	518 (52·0%)	527 (53·3%)
	Patient service assistant or hospital maintenance	138 (13·9%)	109 (11·0%)
Site			
	Campo Grande	546 (54·8%)	545 (55·1%)
	Rio de Janeiro	291 (29·2%)	293 (29·6%)
	Manaus	159 (16·0%)	151 (15·3%)

Data are n (%) or median (IQR).

**Figure 2:**
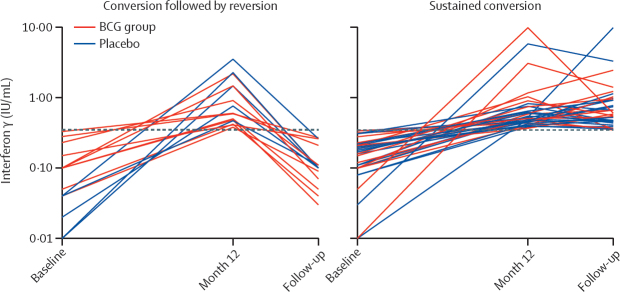
Longitudinal interferon γ values by groups over time in individuals with QFT Plus conversion Longitudinal interferon γ values of the 49 participants (n=25 in BCG group; n=24 in placebo group) at baseline, month 12, and follow-up 6 to 12 months after month 12. Each line represents data for one participant; not shown are data for participants who did not have a QFT Plus conversion and those who had missing QFT results after the initial conversion. The dashed line denotes the manufacturer's recommended threshold for a positive test (≥0·35 IU/mL). QFT=QuantiFERON TB Gold.

During the first 12 months, QFT Plus conversion occurred in 66 (3·3%) of 1985 participants, with no significant differences by study site (p=0·897). Specifically, 34 (3·4%) of 996 participants had initial QFT conversion in the BCG group compared with 32 (3·2%) of 989 in the placebo group (RR 1·09 [95% CI 0·67–1·77]; p=0·791; table 2). Among participants with QFT Plus conversion by 12 months, 25 (74%) of 34 in the BCG group and 24 (75%) of 32 in the placebo group had a follow-up QFT Plus done at least 6 months later (figure 1). Overall, 15 (1·5%) of 996 had a sustained QFT Plus conversion in the BCG group and 19 (1·9%) of 989 in the placebo group (RR 0·80 [95% CI 0·41–1·57]; p=0·510; figure 2).

**Table 2:** Outcomes by study group*

	**BCG group**	**Placebo group**	**Risk ratio (95% CI)**	**p value**
**Primary outcome**				
QFT conversion (positivity threshold ≥0·35 IU/mL)	34/996 (3·4%)	32/989 (3·2%)	1·09 (0·67–1·77)	0·791
**Secondary outcome**				
Sustained conversion (positivity threshold ≥0·35 IU/mL)	15/996 (1·5%)	19/989 (1·9%)	0·80 (0·41–1·57)	0·510
**Exploratory outcomes**				
QFT conversion (positivity threshold ≥0·70 IU/mL)	15/996 (1·5%)	13/989 (1·3%)	1·15 (0·55–2·45)	0·713
QFT conversion (positivity threshold ≥2·00 IU/mL)	6/996 (0·6%)	5/989 (0·5%)	1·18 (0·36–3·85)	0·788
QFT conversion (positivity threshold ≥4·00 IU/mL)	2/996 (0·2%)	3/989 (0·3%)	0·64 (0·11–3·84)	0·633
QFT conversion (baseline QFT <0·20 IU/mL and positivity threshold ≥0·35 IU/mL)	22/957 (2·3%)	23/950 (2·4%)	0·96 (0·53–1·74)	0·903

Data are n/N (%), unless otherwise specified. QFT=QuantiFERON-TB Gold.

* All analyses were done in the intention-to-treat population.

When using an alternative positivity threshold of 0·7 IU/mL or more, 15 (1·5%) of 996 conversions were observed in the BCG group and 13 (1·3%) of 989 in the placebo group (RR 1·15 [95% CI 0·55–2·45]; p=0·713). The BCG group had six (0·6%) of 996 conversions with a positivity threshold of 2·0 IU/mL or more, whereas the placebo group had five (0·5%) of 989 conversions (RR 1·18 [95% CI 0·36–3·85]; p=0·788). Furthermore, QFT Plus conversion in those with a baseline QFT Plus of 0·2 IU/mL with a positivity threshold of 0·35 IU/mL or more occurred in 22 (2·3%) of 957 in the BCG group and 23 (2·4%) of 950 in the placebo group (RR 0·96 [95% CI 0·53–1·74]; p=0·903).

Additional analysis of outcomes in the per-protocol population, encompassing individuals who were allocated with negative QFT Plus at baseline, and presenting results for both those who underwent the month 12 QFT Plus test and those who did not, along with the secondary analyses, is detailed in appendix 2 (pp 5–8).

## Discussion

In this nested randomised controlled trial, we evaluated the efficacy of BCG vaccination in preventing *M tuberculosis* infection, as measured by QFT Plus responses, in adult health-care workers from three sites in Brazil. Most participants had been previously vaccinated with BCG, so the majority assigned to the BCG group were being revaccinated. We found that the BCG-Denmark vaccine did not prevent either QFT Plus conversion by 12 months or sustained QFT Plus conversion compared with placebo. Although event frequency was low, we did not find evidence for protection against QFT Plus conversion infection using any of the study definitions. BCG-Denmark, a vaccine prequalified by WHO, was selected because of its widespread global use. Although variations in the protective efficacy of different BCG vaccine strains have been proposed, this aspect remains unexplored in the context of BCG revaccination. Notably, studies on BCG revaccination have used distinct BCG strains, such as BCG-Moreau in Brazil,^13^ BCG-Glaxo in Malawi,^12^ and BCG-Denmark in South Africa.^11^ Further investigation is warranted to understand the potential effect of BCG strain variability.

The results of this trial add to the mixed evidence concerning the efficacy of BCG revaccination for the prevention of tuberculosis infection or disease. In an open-label, cluster-randomised trial, with BCG revaccination and no placebo done in Brazil with adolescents aged 7–14 years, BCG revaccination showed efficacy of 9% (95% CI –16 to 29) over 5 years^22^ and 12% (–2 to 24) in 9 years of follow-up.^13^ However, in one of the study cities (Salvador), modest efficacy (19% [95% CI 3 to 33]) was reported, whereas in the other trial city (Manaus) no evidence of protection was reported (1% [–27 to 27]).^13^

In Malawi, a double-blind, randomised, placebo-controlled trial of BCG revaccination, including more than 46 000 participants aged between 3 months and 70 years, found no significant overall protection against confirmed tuberculosis infection after 6–9 years of follow-up (incidence rate ratio 1·43).^23^ In addition, in the 30-year follow-up, this population was concluded to have a high HIV prevalence and no evidence for a strong protective effect of repeat BCG against all forms of tuberculosis. However, some evidence exists for modest protection against HIV-negative tuberculosis and protection when the second vaccine was given in childhood.^12^

By contrast, in a trial done in South Africa^11^ in adolescents aged between 12 years and 17 years with an initial negative QFT test, although BCG revaccination did not show efficacy in preventing initial QFT conversion, sustained QFT conversion was reduced by 45·4% (p=0·03) compared with placebo. The event rate in the placebo group was considerably higher in the South African study (15·8% QFT conversion at 24 months) than in our study (3·2% at 12 months), as expected from the higher tuberculosis incidence and burden of infection in South Africa than in Brazil. Further, the 6-month reversion probability following conversion in the placebo group was 20·8% compared with 24·5% in the South African study, which could point towards either a lower average exposure dose or a higher proportion of false-positive conversions in our study. The latter is a possibility, given that the positive predictive value for a QFT conversion is lower in lower incidence settings, as evidenced by the very high reversion rates (>75%) observed in health-care workers in the USA.^24^, ^25^ However, the proportion reverting in our trial and based on very small numbers (n=49), although not statistically significant, was twice as high in the BCG group (ten [40%] of 25) than in the placebo group (five [21%] of 24). The South African trial found that the difference between the groups was primarily driven by reversions in the BCG group, leading the authors to postulate that the BCG effects were primarily through enhancing mycobacterial infection control.

Several plausible explanations exist for the contrasting findings between our study and the South African study. Despite both countries having substantial tuberculosis burden, exposure rates are much higher in the South African communities where that trial was done, as evidenced by lower QFT conversion rates in Brazil. In lower transmission settings, conversions are more likely to be false positives, which could bias findings towards the null. Additionally, studies have suggested that BCG efficacy might vary according to latitude or non-tuberculous mycobacteria exposure.^8^, ^26^, ^27^

The South African trial assessed the efficacy of BCG revaccination in preventing both conversion and sustained conversion, revealing a 50% protection rate supported by 80% statistical power.^11^ Our findings, indicating a 50% protection rate in the BCG group (1·6%) for conversion at 12 months compared with the placebo group (3·2%), correspond to a statistical power of 64·4%. Although this substudy was not originally designed to evaluate tuberculosis prevention through BCG vaccination, its statistical power is not unduly unfavourable.

Previous studies have shown that high IFN-γ concentrations (≥4·0 IU/mL) predict stable conversion and increased risk of tuberculosis infection in infants and adults.^20^, ^21^ The South African trial found a 45·1% reduction in QFT conversions of more than 4·0 IU/mL, similar to the findings against sustained QFT conversion. In our study, only two (0·2%) of 995 participants had conversions of more than 4·0 IU/mL, precluding comparison of this endpoint.

Our study has some limitations. We did not have detailed information about participant exposure to patients with tuberculosis, which would have been a valuable indicator of infection risk. Another limitation is that 17 (26%) of 66 who converted by 12 months did not undergo QFT Plus follow-up testing. Consequently, this omission contributed to our study having reduced power to assess sustained QFT Plus conversion compared with the South African study.^11^ Furthermore, our per-protocol analyses and all analyses restricted to individuals without missing data have the potential for selection biases, whereby missing data might have differed by study group. Additionally, we used QFT Plus conversion and sustained conversion as proxies for *M tuberculosis* infection, consistent with other studies. However, IFN-γ release assays only measure T-cell sensitisation to *M tuberculosis* antigens, and studies have shown both imperfect sensitivities in active disease^28^, ^29^ and high rates of presumed false-positive conversions in populations at low risk.^25^, ^30^ These assays might not be a reliable measure of an individual's current infection status. Finally, the determination of previous BCG status relied on self-reported information, which introduces the possibility of misclassification. However, BCG coverage among health-care workers in Brazil is high. Unfortunately, data regarding BCG vaccination timing were unavailable, and our exclusion criteria only applied to individuals who had received BCG vaccination in the previous year.

In conclusion, our trial done in a high-tuberculosis burden country showed that BCG-Denmark vaccination did not reduce the risk of initial or sustained conversion in adult health-care workers. These results add evidence to the uncertain role of BCG revaccination in adults for the prevention of tuberculosis infection, and current data do not support its use for this indication.

## Data sharing

After the database undergoes cleaning and is finalised, it will be stored in a data-sharing repository archiving system. Access to the data will adhere to the regulations and procedures established by the repository system. Access to the clinical research data package for the BRACE trial will be granted in April, 2025, and is currently under embargo. Requests for data access should be submitted through the Vivli platform using the digital object identifier: https://doi.org/10.25934/PR00007547. The final decision regarding the sharing of this study package will be made by the Welcome Trust Independent Review Panel.

## Declaration of interests

We declare no competing interests.

## Supplementary Materials

Portuguese translation of the abstract
