# Supplementary material for: Effect of BCG vaccination against Mycobacterium tuberculosis infection in adult Brazilian health-care workers: a nested clinical trial
Source: Lancet Infect Dis. 2024 Jun;24(6):594–601. doi: 10.1016/S1473-3099(23)00818-6 (PMC11111441; doi:10.1016/S1473-3099(23)00818-6)
Supplement: Portuguese translation of the abstract [file mmc1.pdf]

THE LANCET
Infectious Diseases

# Supplementary appendix 1

This translation in Portuguese was submitted by the authors and we reproduce it as supplied. It has not been peer reviewed. *The Lancet*'s editorial processes have only been applied to the original in English, which should serve as reference for this manuscript.

Esta tradução em português foi submetida pelos autores e nós não fizemos quaisquer alterações. Esta versão não foi revista por pares. O processo editorial do The Lancet só foi aplicado à versão original em inglês, que deve servir como referência para este artigo.

Supplement to: dos Santos PCP, Messina NL, de Oliveira RD, et al. Effect of BCG vaccination against *Mycobacterium tuberculosis* infection in adult Brazilian health-care workers: a nested clinical trial. *Lancet Infect Dis* 2024; published online Feb 26. https://doi.org/10.1016/S1473-3099(23)00818-6.

**Efeito da vacina BCG contra a infecção por *Mycobacterium tuberculosis* em trabalhadores de saúde adultos brasileiros: um ensaio clínico aninhado**

## Resumo

**Contexto:** A eficácia da vacina Bacillus Calmette-Guérin (BCG) para a tuberculose pulmonar em adultos permanece incerta. Neste estudo, objetivamos avaliar o efeito da vacinação com BCG-Dinamarca para prevenir a conversão inicial e sustentada do ensaio de liberação de interferon-gama em trabalhadores de saúde brasileiros.

**Métodos**: Este subestudo é parte de um ensaio clínico randomizado, o estudo BRACE (NCT04327206), que avaliou a vacina BCG-Dinamarca em trabalhadores de saúde brasileiros (com idade ≥18 anos) de Manaus, Campo Grande e Rio de Janeiro, sem considerar se já haviam sido vacinados com BCG. Foram excluídos os participantes com contraindicações à vacina BCG, tratamento prolongado (>1 mês) com medicamentos antituberculose, reações adversas prévias ao BCG, vacinação recente com BCG ou não adesão às intervenções propostas. Os elegíveis foram alocados aleatoriamente (1:1) para receber uma injeção intradérmica de 0,1 mL de BCG-Dinamarca [cepa dinamarquesa 1331; AJ Vacinas, Copenhague] ou de solução salina a 0,9%, usando um sistema web de randomização em blocos de tamanhos variados (2, 4 ou 6), estratificado por local do estudo, faixa etária (<40, ≥40 a <60, ≥60 anos) e comorbidades (diabetes, doença respiratória crônica, condição cardíaca e hipertensão). Seringas obliteradas evitaram a revelação das atribuições do grupo. O teste QuantiFERON-TB Gold Plus (QFT) Plus (Qiagen; Hilden, Alemanha) foi usado para medir a infecção por tuberculose nas avaliações basais e de 12 meses. O desfecho primário de eficácia foi a conversão do QFT Plus (≥0,35 UI/mL) em 12 meses nos participantes com resultado basal negativo (<0,35 UI/mL).

**Resultados:** Entre 07 de outubro de 2020 e 12 de abril de 2021, 1985 (77,3%) dos 2568 participantes foram elegíveis para a avaliação QFT Plus de 12 meses e foram incluídos neste subestudo; 996 (50,2%) dos 1985 estavam no grupo BCG e 989 (49,8%) estavam no grupo placebo. No geral, 1475 (74,3%) dos 1985 participantes eram mulheres e 510 (25,7%) eram homens, e a idade média era de 39 anos (IQR 32–47). Durante os primeiros 12 meses, a conversão QFT Plus ocorreu em 66 (3,3%) dos 1985 participantes, sem diferenças significativas por local do estudo (p=0,897). Especificamente, 34 (3,4%) dos 996 participantes tiveram conversão QFT inicial no grupo BCG em comparação com 32 (3,2%) dos 989 no grupo placebo (razão de risco 1,09 [IC 95% 0,67–1,77]; p=0,791).

**Interpretação:** A vacinação com BCG-Dinamarca não reduziu o risco de conversão inicial do QFT Plus em trabalhadores de saúde brasileiros. Este achado destaca a necessidade de melhor compreender a prevenção da tuberculose em populações de alto risco.

**Financiamento:** A Bill & Melinda Gates Foundation, a Minderoo Foundation, a Sarah and Lachlan Murdoch, a Royal Children's Hospital Foundation, a Health Services Union NSW, a Peter Sowerby Foundation, a SA Health, a Insurance Advisernet Foundation, a NAB Foundation, a

Calvert-Jones Foundation, a Modara Pines Charitable Foundation, a United Health Group Foundation, a Epworth Healthcare, e doadores individuais.
