# Supplementary material for: Effect of BCG vaccination against Mycobacterium tuberculosis infection in adult Brazilian health-care workers: a nested clinical trial
Source: Lancet Infect Dis. 2024 Jun;24(6):594–601. doi: 10.1016/S1473-3099(23)00818-6 (PMC11111441; doi:10.1016/S1473-3099(23)00818-6)
Supplement: Supplementary appendix 2 [file mmc2.pdf]

THE LANCET
Infectious Diseases

Supplementary appendix 2

This appendix formed part of the original submission and has been peer reviewed. We post it as supplied by the authors.

Supplement to: dos Santos PCP, Messina NL, de Oliveira RD, et al. Effect of BCG vaccination against *Mycobacterium tuberculosis* infection in adult Brazilian health-care workers: a nested clinical trial. *Lancet Infect Dis* 2024; published online Feb 26. https://doi.org/10.1016/S1473-3099(23)00818-6.

Appendix to Effect of BCG vaccination against *Mycobacterium tuberculosis* infection in adult Brazilian health-care workers: a nested clinical trial

Paulo Cesar Pereira dos Santos, M.S.[1], Nicole Messina[2,3], Ph.D., Roberto Dias de Oliveira, Ph.D.[4,5], Patricia Vieira da Silva, Ph.D.[1], Marco Antonio Moreira Puga, Ph.D.[1], Margareth Dalcolmo, Ph.D.[6,7], Glauce dos Santos, B.Sc.[6,7], Marcus Vinícius Guimarães de Lacerda, Ph.D.[8,9], Bruno Araújo Jardim, M.Sc.[8], Fernando Fonseca de Almeida e Val, Ph.D.[8], Nigel Curtis, Ph.D.[2,3,10], Jason Andrews, M.D.[11*], Julio Croda, Ph.D.[1,12,13*]

* These authors contributed equally.

1. Universidade Federal de Mato Grosso do Sul - UFMS, Campo Grande, MS, Brazil.
2. Infectious Diseases Group, Murdoch Children's Research Institute, Parkville, Victoria, Australia.
3. Department of Paediatrics, The University of Melbourne, Parkville, Victoria, Australia.
4. Universidade Estadual de Mato Grosso do Sul, Dourados, MS, Brazil.
5. Programa de Pós-graduação em Ciências da Saúde, Universidade Federal da Grande Dourados, Dourados, MS, Brazil.
6. Centro de Referência Professor Hélio Fraga, Fundação Oswaldo Cruz, Rio de Janeiro, Brazil
7. Pontifícia Universidade Católica do Rio de Janeiro, Rio de Janeiro, Brazil.
8. Fundação de Medicina Tropical Doutor Heitor Vieira Dourado, Manaus, Brazil.
9. Instituto Leônidas & Maria Deane, Oswaldo Cruz Foundation Ministry of Health, Amazonas, Brazil.
10. Infectious Diseases, The Royal Children's Hospital Melbourne, Parkville, Victoria, Australia.
11. Stanford University School of Medicine, Division of Infectious Diseases and Geographic Medicine, Stanford, CA, United States of America.
12. Fiocruz Mato Grosso do Sul, Fundação Oswaldo Cruz, Campo Grande, MS, Brazil.
13. Department of Epidemiology of Microbial Diseases, Yale School of Public Health, New Haven, CT, USA.

Table of Contents

Supplementary Results 2

Supplementary Table 1. The primary analysis of QuantiFERON results stratified by study arm in the intention-to-treat population (regardless of Month 12 QFT performed).* 2

Supplementary Table 2. The secondary analysis of QuantiFERON results stratified by study arm in the intention-to-treat population (Month 12 QFT available).* 3

Supplementary Table 3. The secondary analysis of QuantiFERON results stratified by study arm in the intention-to-treat population (regardless of Month 12 QFT performed).* 4

Supplementary Table 4. The primary analysis of QuantiFERON results stratified by study arm in the per-protocol population (Month 12 QFT available).* 5

Supplementary Table 5. The primary analysis of QuantiFERON results stratified by study arm in the per-protocol population (regardless of Month 12 QFT performed).* 6

Supplementary Table 6. The secondary analysis of QuantiFERON results stratified by study arm in the per-protocol population (Month 12 QFT available).* 7

Supplementary Table 7. The secondary analysis of QuantiFERON results stratified by study arm in the per-protocol population (regardless of Month 12 QFT performed).* 8

Supplementary Results

Supplementary Table 1. The primary analysis of QuantiFERON results stratified by study arm in the intention-to-treat population (regardless of Month 12 QFT performed).*

	BCG group	Placebo group	RR (95% CI)	p-value
**Primary outcome**				
QFT conversion (positivity threshold ≥0·35 IU/mL)	34/1129 (3·0)	32/1140 (2·8)	1·09 (0·66 to 1·77)	0·727
**Secondary outcome**				
Sustained conversion (positivity threshold ≥0·35 IU/mL)	15/1129 (1·3)	19/1140 (1·7)	0·81 (0·41 to 1·60)	0·546
**Exploratory outcomes**				
QFT conversion (positivity threshold ≥0·70 IU/mL)	15/1129 (1·3)	13/1140 (1·1)	1·17 (0·56 to 2·51)	0·671
QFT conversion (positivity threshold ≥2·00 IU/mL)	6/1129 (0·5)	5/1140 (0·4)	1·21 (0·37 to 3·95)	0·757
QFT conversion (positivity threshold ≥4·00 IU/mL)	2/1129 (0·2)	3/1140 (0·3)	0·66 (0·11 to 3·97)	0·654
QFT conversion (baseline QFT <0·20 IU/mL, and positivity threshold ≥0·35 IU/mL)	22/1078 (2·0)	23/1093 (2·1)	0·99 (0·55 to 1·78)	0·971

Data are n/N (%), unless otherwise specified. *All analyses were done in the intention-to-treat population. QFT=QuantiFERON-TB Gold.

Supplementary Table 2. The secondary analysis of QuantiFERON results stratified by study arm in the intention-to-treat population (Month 12 QFT available).*

	BCG group	Placebo group	RR (95% CI)	p-value
**Primary outcome**				
QFT conversion (positivity threshold ≥0·35 IU/mL)	34/996 (3·4)	32/989 (3·2)	1·05 (0·65 to 1·71)	0·833
**Secondary outcome**				
Sustained conversion (positivity threshold ≥0·35 IU/mL)	15/996 (1·5)	19/989 (1·9)	0·79 (0·40 to 1·56)	0·490
**Exploratory outcomes**				
QFT conversion (positivity threshold ≥0·70 IU/mL)	15/996 (1·5)	13/989 (1·3)	1·14 (0·54 to 2·40	0·727
QFT conversion (positivity threshold ≥2·00 IU/mL)	6/996 (0·6)	5/989 (0·5)	1·14 (0·35 to 3·75)	0·825
QFT conversion (positivity threshold ≥4·00 IU/mL)	2/996 (0·2)	3/989 (0·3)	0·62 (0·10 to 3·73)	0·611
QFT conversion (baseline QFT <0·20 IU/mL, and positivity threshold ≥0·35 IU/mL)	22/957 (2·3)	23/950 (2·4)	0·95 (0·53 to 1·72)	0·873

Data are n/N (%), unless otherwise specified. *All analyses were done in the intention-to-treat population. QFT=QuantiFERON-TB Gold.

Supplementary Table 3. The secondary analysis of QuantiFERON results stratified by study arm in the intention-to-treat population (regardless of Month 12 QFT performed).*

	BCG group	Placebo group	RR (95% CI)	p-value
**Primary outcome**				
QFT conversion (positivity threshold ≥0·35 IU/mL)	34/1129 (3·0)	32/1140 (2·8)	1·08 (0·66 to 1·75)	0·762
**Secondary outcome**				
Sustained conversion (positivity threshold ≥0·35 IU/mL)	15/1129 (1·3)	19/1140 (1·7)	0·81 (0·41 to 1·60)	0·551
**Exploratory outcomes**				
QFT conversion (positivity threshold ≥0·70 IU/mL)	15/1129 (1·3)	13/1140 (1·1)	1·17 (0·56 to 2·46)	0·681
QFT conversion (positivity threshold ≥2·00 IU/mL)	6/1129 (0·5)	5/1140 (0·4)	1·17 (0·36 to 3·85)	0·792
QFT conversion (positivity threshold ≥4·00 IU/mL)	2/1129 (0·2)	3/1140 (0·3)	0·64 (0·11 to 3·86)	0·634
QFT conversion (baseline QFT <0·20 IU/mL, and positivity threshold ≥0·35 IU/mL)	22/1078 (2·0)	23/1093 (2·1)	0·98 (0·54 to 1·76)	0·940

Data are n/N (%), unless otherwise specified. *All analyses were done in the intention-to-treat population. QFT=QuantiFERON-TB Gold.

Supplementary Table 4. The primary analysis of QuantiFERON results stratified by study arm in the per-protocol population (Month 12 QFT available).*

	BCG group	Placebo group	RR (95% CI)	p-value
**Primary outcome**				
QFT conversion (positivity threshold ≥0·35 IU/mL)	34/995 (3·4)	32/985 (3·2)	1·06 (0·66 to 1·73)	0·799
**Secondary outcome**				
Sustained conversion (positivity threshold ≥0·35 IU/mL)	15/995 (1·5)	19/985 (1·9)	0·80 (0·40 to 1·57)	0·504
**Exploratory outcomes**				
QFT conversion (positivity threshold ≥0·70 IU/mL)	15/995 (1·5)	13/985 (1·3)	1·15 (0·54 to 2·45)	0·716
QFT conversion (positivity threshold ≥2·00 IU/mL)	6/995 (0·6)	5/985 (0·5)	1·17 (0·36 to 3·85)	0·790
QFT conversion (positivity threshold ≥4·00 IU/mL)	2/995 (0·2)	3/985 (0·3)	0·64 (0·11 to 3·84)	0·632
QFT conversion (baseline QFT <0·20 IU/mL, and positivity threshold ≥0·35 IU/mL)	22/956 (2·3)	23/946 (2·4)	0·96 (0·53 to 1·73)	0·896

Data are n/N (%), unless otherwise specified. *All analyses were done in the per-protocol population. QFT=QuantiFERON-TB Gold.

Supplementary Table 5. The primary analysis of QuantiFERON results stratified by study arm in the per-protocol population (regardless of Month 12 QFT performed).*

	BCG group	Placebo group	RR (95% CI)	p-value
**Primary outcome**				
QFT conversion (positivity threshold ≥0·35 IU/mL)	34/1127 (3·0)	32/1136 (2·8)	1·09 (0·67 to 1·77)	0·731
**Secondary outcome**				
Sustained conversion (positivity threshold ≥0·35 IU/mL)	15/1127 (1·3)	19/1136 (1·7)	0·81 (0·41 to 1·60)	0·542
**Exploratory outcomes**				
QFT conversion (positivity threshold ≥0·70 IU/mL)	15/1127 (1·3)	13/1136 (1·1)	1·17 (0·56 to 2·50)	0·672
QFT conversion (positivity threshold ≥2·00 IU/mL)	6/1127 (0·5)	5/1136 (0·4)	1·20 (0·37 to 3·95)	0·758
QFT conversion (positivity threshold ≥4·00 IU/mL)	2/1127 (0·2)	3/1136 (0·3)	0·66 (0·11 to 3·96)	0·654
QFT conversion (baseline QFT <0·20 IU/mL, and positivity threshold ≥0·35 IU/mL)	22/1076 (0·2)	23/1089 (0·3)	0·99 (0·55 to 1·78)	0·968

Data are n/N (%), unless otherwise specified. *All analyses were done in the per-protocol population. QFT=QuantiFERON-TB Gold.

Supplementary Table 6. The secondary analysis of QuantiFERON results stratified by study arm in the per-protocol population (Month 12 QFT available).*

	BCG group	Placebo group	RR (95% CI)	p-value
**Primary outcome**				
QFT conversion (positivity threshold ≥0·35 IU/mL)	34/995 (3·4)	32/985 (3·2)	1·05 (0·65 to 1·71)	0·842
**Secondary outcome**				
Sustained conversion (positivity threshold ≥0·35 IU/mL)	15/995 (1·5)	19/985 (1·9)	0·79 (0·40 to 1·55)	0·339
**Exploratory outcomes**				
QFT conversion (positivity threshold ≥0·70 IU/mL)	15/995 (1·5)	13/985 (1·3)	1·14 (0·54 to 2·40)	0·731
QFT conversion (positivity threshold ≥2·00 IU/mL)	6/995 (0·6)	5/985 (0·5)	1·14 (0·35 to 3·74)	0·827
QFT conversion (positivity threshold ≥4·00 IU/mL)	2/995 (0·2)	3/985 (0·3)	0·62 (0·10 to 3·72)	0·610
QFT conversion (baseline QFT <0·20 IU/mL, and positivity threshold ≥0·35 IU/mL)	22/956 (2·3)	23/946 (2·4)	0·95 (0·53 to 1·71)	0·867

Data are n/N (%), unless otherwise specified. *All analyses were done in the per-protocol population. QFT=QuantiFERON-TB Gold.

Supplementary Table 7. The secondary analysis of QuantiFERON results stratified by study arm in the per-protocol population (regardless of Month 12 QFT performed).*

	BCG group	Placebo group	RR (95% CI)	p-value
**Primary outcome**				
QFT conversion (positivity threshold ≥0·35 IU/mL)	34/1127 (3·0)	32/1136 (2·8)	1·08 (0·66 to 1·75)	0·768
**Secondary outcome**				
Sustained conversion (positivity threshold ≥0·35 IU/mL)	15/1127 (1·3)	19/1136 (1·7)	0·81 (0·41 to 1·60)	0·545
**Exploratory outcomes**				
QFT conversion (positivity threshold ≥0·70 IU/mL)	15/1127 (1·3)	13/1136 (1·1)	1·17 (0·56 to 2·46)	0·682
QFT conversion (positivity threshold ≥2·00 IU/mL)	6/1127 (0·5)	5/1136 (0·4)	1·17 (0·36 to 3·84)	0·794
QFT conversion (positivity threshold ≥4·00 IU/mL)	2/1127 (0·2)	3/1136 (0·3)	0·64 (0·11 to 3·85)	0·633
QFT conversion (baseline QFT <0·20 IU/mL, and positivity threshold ≥0·35 IU/mL)	22/1076 (0·2)	23/1089 (0·3)	0·98 (0·54 to 1·76)	0·936

Data are n/N (%), unless otherwise specified. *All analyses were done in the per-protocol population. QFT=QuantiFERON-TB Gold.
